# The relationship between the level of glutathione, impairment of glucose metabolism and complications of diabetes mellitus

**DOI:** 10.12669/pjms.294.2859

**Published:** 2013

**Authors:** Ismail Hakki Kalkan, Murat Suher

**Affiliations:** 1 Ismail Hakki Kalkan, MD, Department of Gastroentorology, Kirikkale State Hospital, Kirikkale, Turkey.; 2 Murat Suher, MD, Associate Professor, Department of Internal Medicine, Ataturk Training and Research Hospital, Ankara, Turkey.

**Keywords:** Complications, Diabetes mellitus, Glutathion

## Abstract

***Objective:*** To investigate whether there is a difference between the subjects with new-onset type 2 diabetes mellitus (DM), impaired glucose tolerance (IGT) and normal fasting blood glucose levels with respect to the level of glutathione (GSH) and the relationship between the presence of complication of diabetes and the level of GSH.

***Methods:*** Oral Glucose Tolerance Test (OGTT) was performed in IFG patients, with no episode of drug use, who were admitted to hospital. According to the results of the application 30 subjects with type 2 DM, 30 subjects with IGT and 28 subjects with normal blood glucose level were included in the study. Anthropometric measurements and blood pressure values of all subjects were recorded. The biochemical parameters of subjects were studied in the biochemistry laboratory by utilizing Olympus AV-2700. The subjects with diabetic retinopathy and nephropathy were established subsequent to the examination of the retina and 24-hour urine collection test performed to subjects with diagnosis of DM. Levels of GSH in all subjects were measured by enzymatic recycling method.

***Results:*** The mean levels of GSH in subjects with DM were significantly reduced compared with IGT or normal subjects (respectively p=0.02 and p<0.001). Besides, lower levels of GSH were acquired in subjects with IGT compared to normal subjects (p<0.001). The mean levels of GSH in subjects with diabetic retinopathy were lower than the subjects with no established diagnosis of diabetic retinopathy (p<0.001). Similarly, lower levels of GSH (p<0.001) were obtained in microalbuminuric subjects than normoalbuminuric subjects.

***Conclusions:*** At the end of the study, we came to the conclusion that GSH deficiency was of great significance in the pathogenesis of Diabetes Mellitus.

## INTRODUCTION

Oxidative stress developing due to the chronic hyperglycemia play an important role in the etiology of diabetic complications. Chronic hyperglycemia causes increase in the activity of polyol pathway and the rate of NADH/NAD. The imbalance in the reduction reaction is related to the occurrence of partially accelerated oxidation of sorbitol to fructose via NAD related sorbitol dehydrogenase, and partially increased free radicals.^1^^,^^2^

Glutathione (GSH), an intracellular thiol causes the eradication of free radicals or reduction in hydrogen peroxide level on state of oxidative stress. Decrease in the reduced GSH level and impairment in GSH metabolism have been reported in the erythrocyte of diabetics. Decrease in the level of GSH occurs both due to the competition between aldose reductase and glutathione reductase for NADPH, a cofactor, and increased oxidative stress (increased ratio of NADH/NAD).^3^

We aimed to investigate whether there is a difference between the subjects with new-onset type 2 DM, impaired glucose tolerance (IGT) and normal fasting blood glucose levels. Moreover, we studied the correlation between the presence of complication of diabetes and the value of GSH in subjects with diabetes mellitus (DM).

## METHODS

The study group consisted of 30 subjects with type 2 DM, 30 subjects with IGT according to the results obtained in the OGTT and 28 subjects with normal blood glucose values and no chronic disease comprised the control group. Patients with episodes of drug use for any chronic disease, active infections and smokers were excluded from the study group.

The fasting blood glucose, urea, creatinine, HbA1c, fructosamine were studied by utilizing Olympus AV-2700. Insulin and c-peptide levels were measured by using immune apparatus. Homeostasis Model Assessment-Insulin Resistance (HOMA-IR) values were obtained by the formula: insulin (uIU/ml) x fasting blood glucose (mg/dl)/405.

The subjects were examinated by an ophthalmologist for diabetic retinopathy. The 24-hour urine were collected. Microalbumine value below 30 mg/day were regarded normoalbuminuric, microalbumine value over 30 mg/day were regarded microalbuminuric.

**Studying GSH**

 To quantify GSH levels, the Calbiochem® GSH Assay Kit II was used. This GSH Assay Kit II utilizes a carefully optimized enzymatic recycling method, using glutathione reductase, for the quantification of GSH.

*Dispersion of the Serum*

1. Blood sample was collected into biochemistry tubes containing no anticoagulants. Blood sample was left for coagulation at a temperature of 25 C° for 30 minutes. 

2. Blood sample was centrifuged at 2000 rpm 15 minutes at 4 C°. Yellow layer on the serum was removed.

3. Since the dispersed serums could not be studied on the same day, deproteinization procedure was carried out.

*Deproteination Process:*

1. MPA Reagent: 5g metaphosphoric acid (Aldrich, Cat# 43157-5) was dissolved in 50 ml water.
2. Serum sample was mixed with an equal volume of the MPA reagent. The mixture was allowed to stand at room temperature for 5 min and centrifuged at 2000 rpm for approximately two minutes. The supernatant was stored at -20°C.
3. TEAM reagent: 531µl triethanolamine (Aldrich, Cat# T5830-0) was mixed with 469 µl water to prepare 4M TEAM reagent. 50 µl of TEAM reagent per ml of the supernatant was added and vortexed immediately.

*Plate Configuration:*

1. Preparation of the standards: 50 µl of standard was added to 8 wells on the plate.
2. 50 µl of sample was added to each of the sample wells.
3. The plate was covered with the plate cover provided.
4. Assay Cocktail was prepared by mixing the following reagents:

a) 11.25 ml MES Buffer consists of 0.4 M 2-(N-morpholino) ethanosulphonic acid, 0.1 M phosphate, and 2 mM EDTA.)

b) 0.45 ml reconstituted Cofactor Mixture

c) 2.1 ml reconstituted Enzyme Mixture

d) 2.3 ml water

e) 0.45 ml reconstituted DTNB ((5,5’-dithiobis-2-nitrobenzoic)

1. 150 µl of the freshly prepared Assay Cocktail after the removal of the plate cover was added to each of the wells containing standards and samples.
2. The plate cover was replaced and the plate was incubated in the dark on an orbital shaker.
3. Absorbance in the wells were measured at 405 nm for 25 minutes system using a plate reader
4. Calculating the Results: GSH concentration of the samples was determined by the End Point Method.

Descriptive statistics for all variables were made. One-Way ANOVA test was used to compare GSH levels, BMI, BFI, WHR and cyctolic/diastolic blood pressures among the impairment of glucose metabolism groups. Comparison of GSH levels according to the gender in all impairment of glucose metabolism groups, diabetic complication groups, fructosamine and HbA1C level groups was calculated with Student T test. Chi square (χ2) test was used to compare gender distribution between impairment of glucose metabolism groups. The correlation between GSH levels and other biochemical variables was calculated using the Pearson test. Ninety-five percent confidence interval was identified in the analysis and two-sided p <0.05 was considered as significant. Statistical Package for Social Sciences (SPSS) 16 Version pocket program was used for statistical analysis.

## RESULTS

A total of 88 subjects were included to the study. Of them 30 (34.1%) were with type 2 DM, 30 (34.1%) were with IGT, but 28 (31.2%) subjects in the control group were normal with respect to the glucose metabolism.

Mean GSH values were 3.9 ± 1.1 µM in subjects with DM, 4.83 ± 1.66 µM in subjects with IGT and 8.37 ± 1.04 µM in normal subjects. There was a statistical difference with respect to mean GSH values between the subjects with DM and IGT and normal subjects, (respectively p=0.02 and p<0,001). Besides, there was a significant difference between the subjects with IGT and normal subjects (p<0,001).

When male and female subjects were compared separately in 3 groups with respect to mean GSH values, the difference was statistically insignificant.

When the correlation between GSH value and age, fasting blood glucose, HbA1c, fructosamine, urea, uric acid, creatinine, HOMA-IR values were analyzed by Pearson’s correlation test, strong negative correlation was determined with fasting blood glucose. Besides, middling negative correlation was found between GSH value and HbA1c, fructosamine, urea and HOMA-IR values.

HbA1c values were above 7 in 7 (23.3%) of the DM determined subjects; whereas, they were below 7 in 23 (76.7%) subjects. Statistically significant differences were obtained between the subjects with HbA1c values above and below 7 with respect to GSH values (p<0.001).

Increased fructosamine values were determined in 11 (36.7%) of the subjects with DM. The mean GSH values of DM subjects with increased fructosamine values were significantly lower than that of the normal subjects (p=0.002).

**Table-I:** The correlation between HbA1c and mean GSH, fructosamine and mean GSH in subjects with DM

	*Mean GSH (M)*	*P Value*
HbA1c		
<7	4.2 ± 1.1	<0.001
>7	2.9 ± 0.1	
Fructosamine		
Normal	4.3 ± 1.1	0.002
Increased	3.1 ± 0.6	

Diabetic retinopathy was present in 4 (13.3%) subjects with DM and the examination of the retina of 26 (86.7%) subjects was normal. There was a significant difference (p<0.001) with respect to mean GSH values between the diabetic retinopathy determined subjects and diabetic retinopathy was not determined.

**Table-II:** The correlation between diabetic retinopathy and mean GSH

	*n (%)*	*Mean GSH (M)*	*P Value*
Retinopathy			<0.001
Absent	26 (86.7)	4.0 ± 1.1	
Present	4 (13.3)	3.0 ± 1.1	

Statistically significant difference was obtained (p<0.001) when the mean GSH values of 5 (16.7%) subjects with DM, in whom microalbuminuria was determined as the 24-hour urine collection test result, and normoalbuminuric 25 (83.3%) subjects were compared.

**Table-III:** The correlation between the diabetic nephropathy and mean GSH

	*n (%)*	*Mean GSH (M)*	*P Value*
Microalbuminurea			<0.001
Absent	25 (83.3)	4.1 ± 1.1	
Present	5 (16.7)	3.0 ± 0.1	

## DISCUSSION

The presence of oxidative stress in DM has been usually determined by the increase in plasma markers of oxidative stress or decrease in plasma antioxidant levels. Contradictory results in relation with GSH were obtained in various studies.^4^

The antioxidation capacity has been measured in subjects with type 2 DM in two studies. Impairment in the antioxidant capacity of the subjects was determined in one of the studies; however, no difference was determined between the subjects with DM and the control group in the other study.^4^^,^^5^

Different results showing decreased, increased or normal GSH values have been presented.^6^ We obtained lower GSH values in the subjects with DM with respect to subjects with IGT and normal subjects. We compared the subjects with impaired glucose metabolism (DM, IGT) with the mean serum GSH in normal subjects. We determined lower GSH values in the subjects with IGT in comparison to the normal subjects.

The progressive drop in GSH values due to the difference in the anti-oxidant defense during aging is the most frequently reported information. Lang et al determined significant drop in GSH values, independent of sexuality, in healthy aging subjects.^7^ We were not able to evaluate the effects of age to the mean GSH in the groups since we studied with similar age groups. However, no significant result was obtained when the correlation between the average age of all subjects and mean GSH values were examined by using correlation test.

Lower values of GSH were obtained from males in comparison to females in a number of subsequent studies. No difference between GSH values with respect to sexuality was determined in our study.

Hyperglycemia causes an increase in reactive oxygen metabolites and their derivatives.^8^ Since GSH is an important antioxidant, GSH deficiency causes increase in the oxidative stress.^9^ Powell et al showed hyperglycemia induced oxidative stress and reduction in the levels of GSH in the vascular straight muscles.^10^ We determined high value negative correlation between fasting blood glucose and GSH levels when we tested the correlation between the GSH levels and biochemical parameters.

Blood glucose regulation was also indicated to be in relation with GSH values. Lower GSH values were reported to be present in DM subjects with poor follow-up. Seghrouchni et al determined negative correlation between the GSH and HbA1c values in diabetic patients. A similar result was obtained in a study of Giugliano et al.^9^^,^^11^ We determined a negative correlation between HbA1c and fructosamine values, a scale of blood glucose regulation, and GSH values. In addition we determined lower GSH values in diabetic subjects with HbA1c value >7 when compared to <7.

Oxidative stress play an important role in the pathogenesis of microvascular complications.^12^ In some studies the oxidation products of diabetic subjects with chronic complications were determined to be at higher levels than that of the diabetic subjects without complications.^13^ It has been reported that the antioxidant defense system was weakened in subjects with DM.^14^

Oxidative stress markers were observed to increase in mice with early stage diabetic nephropathy when compared to the control group.^15^ There have not been sufficient studies on the direct measurement of the effect of the oxidative stress over the kidneys in humans or the investigations on the effects of antioxidants on albuminuria.^16^ Chiarelli et al determined increase in the markers of oxidative stress together with albumin excretion rate in diabetic subjects.^17^ Beisswenger et al determined a negative correlation between low levels of GSH and glomerular basement membrane thickening in diabetic subjects.^18^ Ozdemir et al. determined lower values of GSH in diabetic subjects with microalbuminuria than normoalbuminuric subjects.^19^ We have determined lower values of GSH in diabetic subjects with microalbuminuria than normoalbuminuric subjects. Development of diabetic retinopathy is accelerated by poor glycemic control and the elevated glucose level increase the superoxide ion.^20^

Increase in the lipid peroxide compounds and reduction in SOD activity and GSH in diabetic mice retina have been displayed.^21^ Hartnett et al determined increase in the markers of oxidative stress and decrease in the antioxidant enzyme levels in subjects with diabetic retinopathy.^22^ The mechanism of reduction in retinal GSH determined in DM has not been clarified yet. It has been indicated that there may be an increase in GSH consumption in DM or reduction of GSH synthesis. In some other studies it has been defended that there is no failure of GSH synthesis in diabetic mice retina; however, there is reduction of enzyme levels in the GSH redox cycle.^23^ The GSH levels of diabetic subjects with diabetic retinopathy were determined significantly lower than those of the diabetic subjects without diabetic retinopathy in our study as well.

## CONCLUSION

In conclusion we have obtained lower GSH values in subjects with new-onset DM than subjects with IGT and normal subjects. Additionally, observation of lower GSH values in subjects with IGT than normal subjects has shown that there is a problem in the anti-oxidative defense mechanism at each stage of glucose metabolism impairment. Obtaining lower GSH values in subjects with diabetic microvascular complications or poor follow-up DM has made us think about these parameters to be an important prognostic factor in subjects with DM. In addition, GSH deficiency will make the present state get worse by causing increase of oxidative stress since GSH is an important antioxidant.

## References

[1] Maritim AC, Sanders RA, Watkins JB (2003). Diabetes, oxidative stress, and antioxidants: a review. J Biochem Mol Toxicol..

[2] Vanderjagt DJ, Harrison JM, Ratliff DM, Hunsaker LA, Vanderjagt DL (2001). Oxidative stress indices in IDDM subjects with and without long-term diabetic complications. Clin Biochem..

[3] Pastore A, Federici G, Bertini E, Piemonte F (2003). Analysis of glutathione: Implication in redox and detoxification. Clin Chim Acta..

[4] Nuttall SL, Dunne F, Kendall MJ, Martin U (1999). Age-independent oxidative stress in elderly patients with non-insulin-dependent diabetes mellitus. QJM..

[5] Opara EC, Abdel-Rahman E, Soliman S (1999). Depletion of total antioxidant capacity in type 2 diabetes. Metabolism..

[6] Seghieri G, Di Simplicio P, Giorgio LA (2000). Relationship between metabolic glycaemic control and platelet content of glutathione and its related enzymes in insulin-dependent diabetes mellitus. Clin Chim Acta..

[7] Lang CA, Naryshkin S, Schneider DL, Mills BJ, Lindeman RD (1992). Low blood glutathione concentrations in healthy aging adults. J Lab Clin Med..

[8] Ceriello A, Giugliano D, Alberti KGMM, Zimmet P, Fronzo RA (2001). Oxidative stress and Diabetic complications. International Textbook of Diabetes Mellitus.

[9] Seghrouchni I, Drai J, Bannier E, Riviere J, Calmard P, Garcia I (2002). Oxidative stress parameters in type 1, type 2 and insulin-treated type 2 diabetes mellitus; insulin treatment efficiency. Clin Chim Acta..

[10] Powell LA, Nally SM, McMaster D, Catherwood MA, Trimble ER (2001). Restoration of glutathione levels in vascular smooth muscle cells exposed to high glucose conditions. Free Radic Biol Med..

[11] Giugliano D, Ceriello A, Paolisso G (1996). Oxidative stress and diabetic vascular complications. Diabetes Care..

[12] Wierusz-Wysocka B, Wysocki H, Byks H, Zozulińska D, Wykretowicz A, Kaźmierczak M (1995). Metabolic control quality and free radical activity in diabetic patients. Diabetes Res Clin Pract..

[13] Bambolkar S, Sainani GS (1995). Evaluation of oxidative stress with or without vascular complications. J Assoc Physicians India.

[14] Rema M, Mohan V, Bhaskar A, Shanmugasundaram KR (1995). Does oxidant stress play a role in diabetic retinopathy?. Indian J Ophtalmol..

[15] Van Dam PS, van Asbeck BS, Erkelens DW, Marx JJM, Gispen WH, Bravenboer B (1995). The role of oxidative stress in neuropathy and other diabetic complications. Diabetes Metab Rev..

[16] Gerstein HC, Bosch J, Pogue J, Taylor DW, Zinman B, Yusuf S (1996). Rationale and design of a large study to evaluate the renal and cardiovascular effects of an ACE inhibitor and vitamin E in high-risk patients with diabetes. Diabetes Care..

[17] Chiarelli F, Di Marzio D, Santilli F, Mohn A, Blasetti A, Cipollone F (2005). Effects of Irbesartan on intracellular antioxidant enzyme expression and activity in adolescents and young adults with early diabetic angiopathy. Diabetes Care..

[18] Beisswenger PJ, Drummond KS, Nelson RG, Howell SK (2005). Susceptibility to diabetic nephropathy is related to dicarbonyl and oxidative stress. Diabetes..

[19] Özdemir G, Özden M, Maral H, Kuskay S, Çetinalp P, Tarkun I (2005). Malondialdehyde, glutathione, glutathione peroxidase and homocysteine levels in type 2 diabetic patients with and without microalbuminuria. Ann Clin Biochem..

[20] Williamson JR, Chang K, Frangos M, Hasan KS, Ido Y, Kawamura T (1993). Hyperglycemic pseudohypoxia and diabetic complications. Diabetes..

[21] Agardh CD, Agardh E, Qian Y, Hultberg B (1998). Glutathione levels are reduced in diabetic rat retina but are not influenced by ischemia followed by recirculation. Metabolism..

[22] Hartnett ME, Stratton RD, Browne RW, Rosner BA, Lanham RJ, Armstrong D (2000). Serum markers of oxidative stress and severity of diabetic retinopathy. Diabetes Care..

[23] Kowluru RA, Kern TS, Engerman, Armstrong D (1996). Abnormalities of retinal metabolism in diabetes or experimental galactosemia.III. Effects of antioxidants. Diabetes..

